# Evaluation of Experience, Training, and Hand Dominance on Drilling Accuracy in Orthopedic Surgeons—A Preliminary Study

**DOI:** 10.3390/medicina62010026

**Published:** 2025-12-23

**Authors:** Etay Elbaz, Nadav Graif, Efi Kazum, Yaniv Warschawski, Jonathan Kleczewski, Asaf Bibas, Ron Gurel, Shai Factor

**Affiliations:** Tel Aviv Medical Center, Department of Orthopedic Surgery, Gray Faculty of Medical & Health Sciences, Tel Aviv University, Weizmann St. 6, Tel Aviv-Yafo 6423906, Israel

**Keywords:** simulation training, clinical competence, hand dominance, orthopedic procedures, drilling

## Abstract

*Background and Objectives*: To evaluate the association of surgeon experience, simulation-based training, and hand dominance on drilling accuracy using a synthetic bone model, with the hypothesis that training improves resident performance and left-handed individuals show superior bilateral accuracy. *Materials and Methods*: A prospective observational study was conducted in the Orthopedic Surgery Division of a tertiary academic center. Drilling accuracy was assessed before and after a standardized simulation-based training program. Twenty-five orthopedic surgeons participated: 9 junior residents (≤3 years of training), 8 senior residents (>3 years), and 8 board-certified experts. All participants completed baseline assessments; only residents were evaluated immediately after training and at a 2-week follow-up. *Results*: Experts showed superior baseline accuracy, particularly with the non-dominant hand. Senior residents showed a significant overall effect of time on right-hand accuracy (F(2,14) = 5.85, *p* = 0.014); post hoc pairwise comparisons showed trends toward improvement from baseline to post-training (*p* = 0.06) and from post-training to 2-week follow-up (*p* = 0.105); Junior residents showed no significant changes. Left-handed participants consistently outperformed right-handed peers with their non-dominant hands (*p* = 0.034). Among residents, this pattern persisted across all sessions. At baseline, senior residents and experts had similar right-hand accuracy (*p* = 0.59), but senior residents performed worse with the left hand (*p* = 0.038). No significant differences were found between junior and senior residents in either hand across all time points, indicating that residency duration alone does not improve performance without targeted training. *Conclusions*: Drilling accuracy in orthopedic surgery is influenced by experience level, targeted training, and hand dominance. Experts show greater precision, and senior residents showed a significant overall effect of time on right-hand accuracy, with trends toward improvement following training, while junior residents may need different training strategies. Tailored educational interventions are needed to improve accuracy and ambidexterity across all training stages. Level of evidence: II.

## 1. Introduction

Accurate bone drilling is a fundamental technical skill in orthopedic surgery, essential for proper implant placement, fracture fixation, and the prevention of complications such as neurovascular injury, implant failure, and delayed healing. Precision in drilling directly influences patient outcomes and requires a high degree of motor control and spatial judgment, skills that are developed through sustained, deliberate practice. Despite its clinical importance, limited quantitative data exists regarding how drilling accuracy is affected by variables such as surgeon experience, technical training, and hand dominance [1,2].

Orthopedic residents typically acquire technical proficiency through hands-on experience in the operating room, where they learn to optimize drill plunge depth, minimize radiation exposure, and develop procedural confidence. However, increasing financial constraints, reduced availability of cadaveric specimens, and work-hour limitations have created significant barriers to traditional surgical training [3]. These challenges underscore the growing need for cost-effective, high-yield simulation models that allow trainees to refine tactile and visuospatial skills in a safe, controlled environment outside the operating room.

Simulation-based training has demonstrated value in enhancing various aspects of surgical technique, including drilling accuracy and depth control. For example, Ruder et al. [1] and Kazum et al. [2] showed that low-cost drilling simulators significantly reduced plunge depth and improved performance in both junior and senior residents. Brichacek et al. [4] further demonstrated the use of 3D-printed models to improve Kirschner wire placement in hand fractures. Moreover, high-fidelity tactile simulators have shown potential in training complex procedures such as closed reduction and percutaneous pinning (CRPP), offering opportunities for repetitive practice and feedback without patient risk. Recent work further highlighted that training benefits are retained over time, with residents demonstrating sustained improvement in drilling accuracy after repeated exposure to structured simulation modules. Collectively, these findings suggest that simulation provides a robust and reproducible framework for technical skill acquisition in orthopedics [5].

An emerging yet underexplored factor in surgical performance is hand dominance. While the broader surgical literature has discussed the challenges encountered by left-handed surgeons, particularly regarding the ergonomic limitations and lack of ambidextrous instrumentation, there is a notable gap in data assessing the impact of handedness on technical performance in orthopedic procedures [6,7]. Approximately 15% of orthopedic surgeons identify as left-hand dominant (LHD), with significantly higher rates of ambidexterity in tasks such as scalpel and suturing use compared to their right-hand dominant (RHD) peers. Although LHD individuals often adapt by developing bimanual skills, few report receiving formal laterality-specific psychomotor training during residency [8]. Some evidence suggests that left-handed surgeons may benefit from targeted training strategies and that simulation may help mitigate their challenges by promoting adaptability and tool familiarity [9].

This study aims to address key gaps in the orthopedic surgical education literature by: (1) quantitatively assessing drilling accuracy across different levels of surgical experience; (2) evaluating the association with a focused, low-cost simulation-based training module; (3) investigating the role of hand dominance in drilling performance, including comparison between dominant and non-dominant hand use.

We hypothesized that simulation-based training would improve drilling accuracy among residents and that left-handed residents, due to their need for bilateral adaptability, might demonstrate superior performance in certain tasks.

## 2. Materials and Methods

### 2.1. Study Design and Participants

This prospective experimental study evaluated differences in drilling accuracy among orthopedic surgeons of varying levels of experience, as well as the association of training and handedness. All participants were recruited from a single orthopedic department and categorized into three groups based on their level of surgical training:

Junior residents: ≤3 years into orthopedic residency;

Senior residents: >3 years into orthopedic residency;

Experts: board-certified orthopedic surgeons.

Each participant self-reported their dominant hand. All participating surgeons voluntarily agreed to take part in the study after receiving a full explanation of its purpose and procedures.

### 2.2. Drilling Protocol

**Figure 1:**
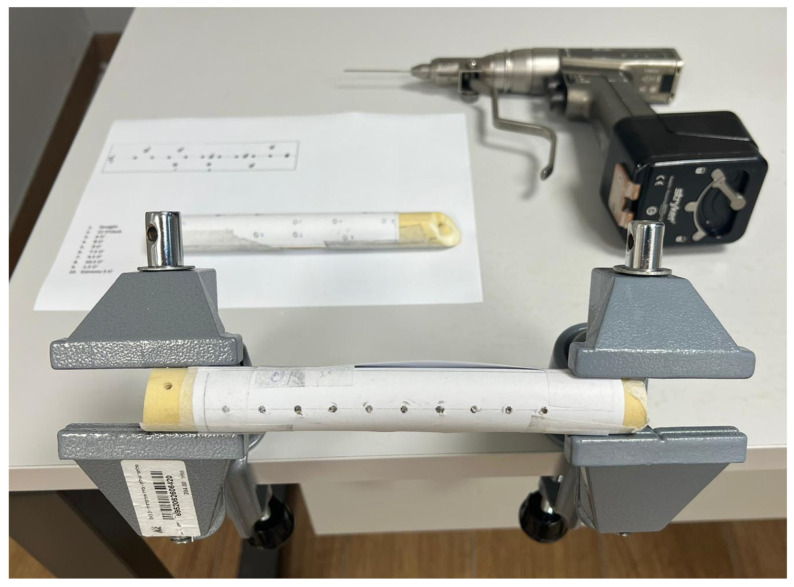
Complete setup used for the study, including the surgical motor, Kirschner wire, instructional and objective sheet, and an artificial Saw bone wrapped in paper indicating entry points. The bone is stabilized on a standard table using general-purpose clamps.

**Figure 2:**
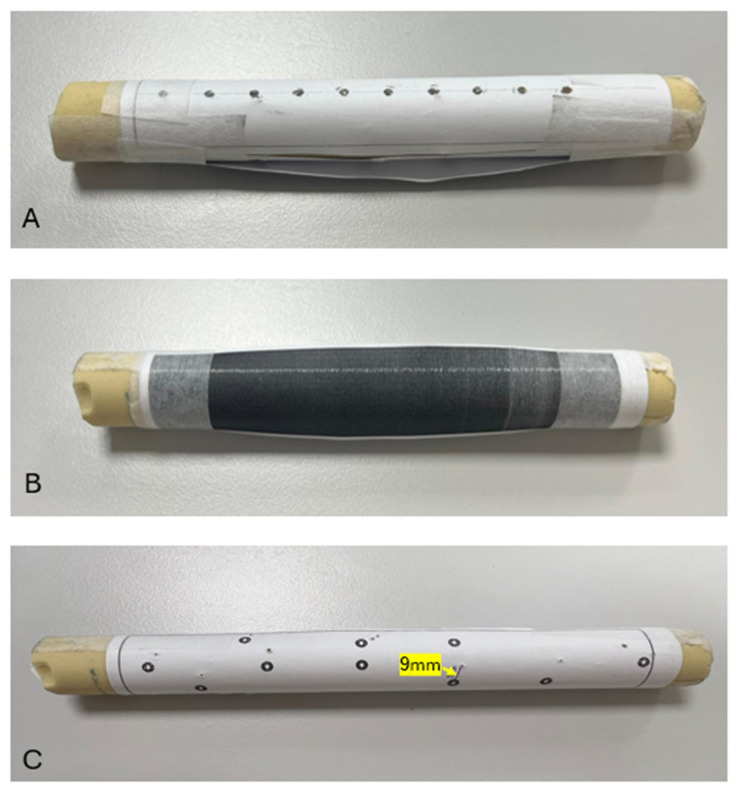
(**A**) Preparation of the Saw bone with 10 marked entry points. (**B**) The exit area is concealed from the participant’s view. (**C**) Desired exit points and the holes created by K-wire insertion.

**Figure 3:**
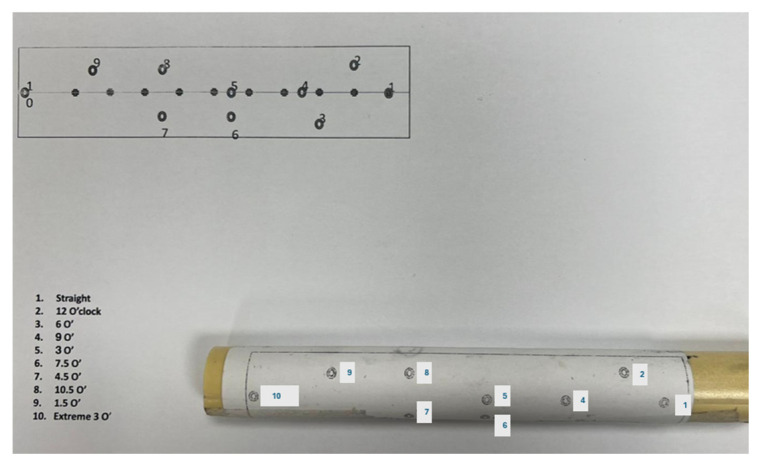
The instructional sheet includes numbered targets, each with a designated directional objective. For example, point number 2 is to be directed vertically upward, corresponding to the 12 o’clock position.

**Figure 4:**
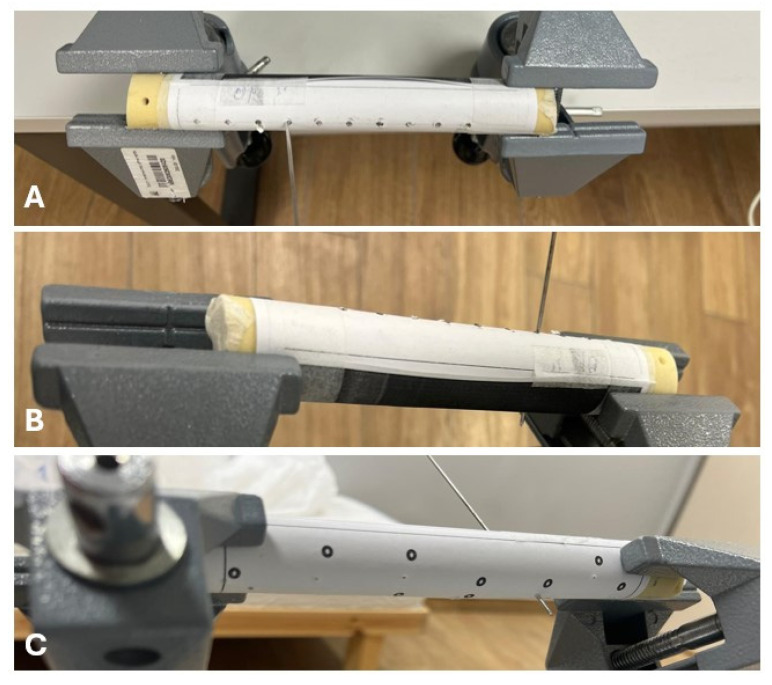
(**A**) Insertion of the K-wire at point number 4, as indicated on the instructional sheet. (**B**) During the procedure, the participant cannot see the outcome, as the exit side is covered with black tape. (**C**) The K-wire and its exit point after removal of the black tape.

**Figure 5:**
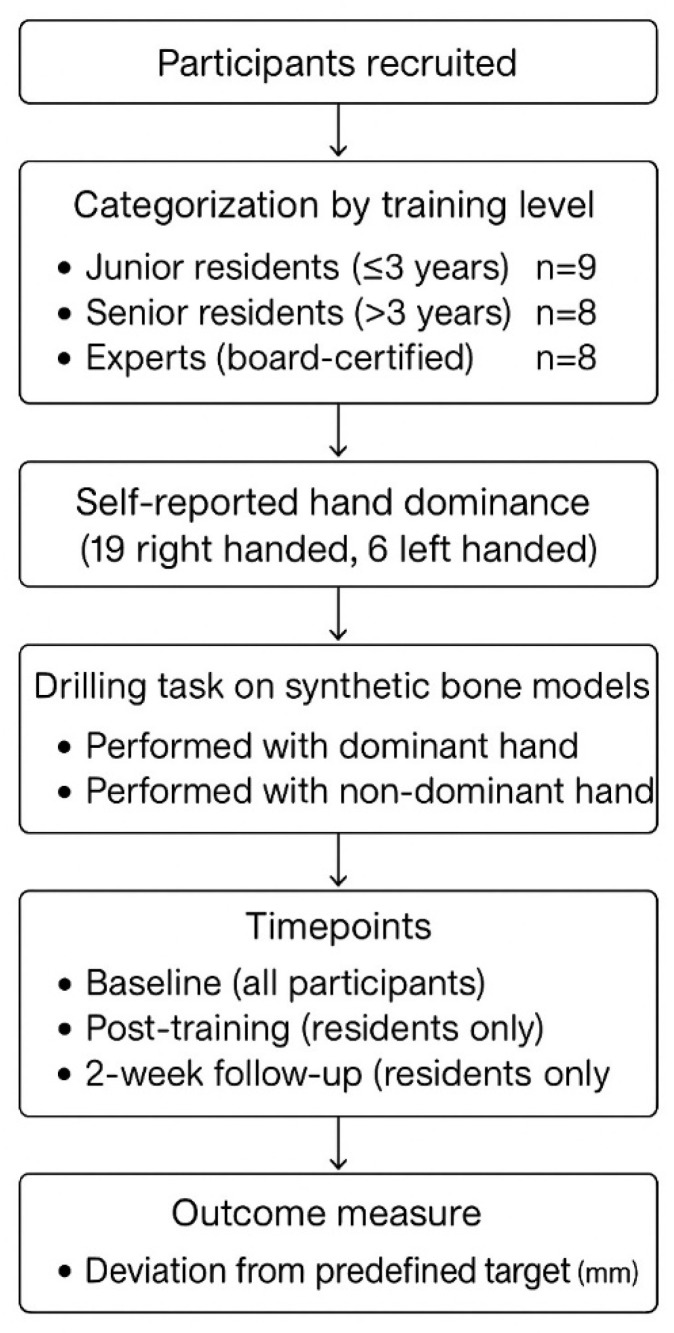
Flowchart of the study design.

Participants performed a series of bone drilling tasks using synthetic bone models in a simulated setting. Drilling was performed using both the dominant and non-dominant hands. All participants completed the baseline assessment, while only residents continued to do two additional time points: immediately post-training and a two-week follow-up. The training session comprised a focused and structured hands-on drilling workshop using synthetic bone models simulating cortical bone density. Beyond simple repetition, the session emphasized deliberate practice aimed at improving technical accuracy. Following the baseline assessment, each participant received individualized feedback from an experienced instructor, identifying specific aspects such as entry angle control and drilling stability. Residents were encouraged to reflect on this feedback and adjust their technique accordingly during the session. The training allowed sufficient time for repeated practice and refinement, aiming to instill core principles of accurate drilling. At each timepoint, participants performed 10 consecutive drills with each hand. For each drilling, deviation from a predefined target point was measured in millimeters. The mean deviation from the intended target and the total time to complete all 10 drillings per hand were recorded. Measurements were performed using a Vernier caliper, with results rounded to one decimal place (0.1 mm) to reflect clinically relevant precision, as shown in Figure 1, Figure 2, Figure 3, Figure 4 and Figure 5.

The synthetic bone models used in this study were selected for their anatomical consistency and standardized cortical density, which approximate human bone characteristics relevant to the drilling task. While formal construct or face validity testing of the simulation system was not performed as part of this study, similar models have been widely adopted in surgical education for practicing drilling techniques [2,10]. Exit point deviation in millimeters was chosen as the primary outcome measure because it represents a quantifiable and clinically relevant indicator of drilling accuracy, with direct implications for surgical safety in procedures involving bicortical fixation or proximity to neurovascular structures. The model enabled controlled conditions that isolated drilling performance, minimizing confounding variables present in the clinical setting.

### 2.3. Data Collection

Data were recorded in a structured Excel file with consistent identifiers for each participant across all timepoints. Deviation from the intended target values were collected for each point (R1–R10 for right hand, L1–L10 for left hand). The mean deviation from the intended target per hand and total task time were calculated and used for analysis.

### 2.4. Statistical Analysis

Descriptive statistics included participant counts, group distribution, handedness, and mean ± standard deviation for drilling accuracy measures. Data distribution was assessed for normality using the Shapiro–Wilk test. Although the formal test results are not reported for each individual measure due to the small sample size, the distributions were approximately normal based on descriptive statistics, supporting the use of parametric tests.

Between-group comparisons at baseline were performed using independent samples *t*-tests. For within-subject comparisons across three time points (Baseline, Post-training, 2 Weeks) in residents, repeated measures analysis of variance (ANOVA) was applied separately for junior and senior residents for each hand. When the overall ANOVA F-test was statistically significant, post hoc pairwise comparisons were conducted using Bonferroni correction to control for multiple comparisons. Statistical significance was defined as *p* < 0.05. All analyses were performed using SPSS Statistics software version 29 (IBM SPSS, New York, NY, USA).

## 3. Results

### 3.1. Participant Demographics

A total of 25 participants were enrolled in the study: 9 junior residents, 8 senior residents, and 8 expert surgeons. The dominant hand distribution included 19 right-handed (76%) and 6 left-handed (24%) individuals across all experience levels. All participants completed the baseline assessment; however, only the 17 residents (junior and senior) completed the post-training session and the 2-week follow-up assessment. Expert surgeons participated only in the baseline assessment (Table 1).

### 3.2. Drilling Accuracy by Experience Level

**Figure 6:**
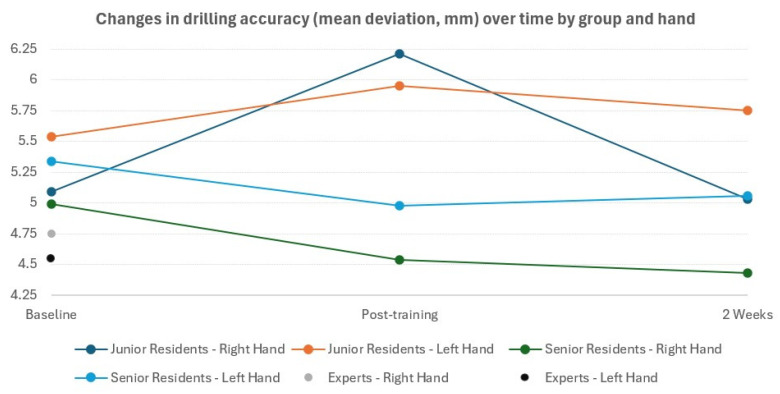
Changes in drilling accuracy (mean deviation, mm) over time by group and hand.

At baseline, expert surgeons demonstrated superior drilling accuracy compared to both junior and senior residents (Figure 6) The expert group’s mean deviation from the intended target (right hand: 4.75 ± 0.91 mm; left hand: 4.55 ± 0.79 mm) were established as the reference standard for comparison. Senior residents showed baseline deviations of 4.99 ± 1.41 mm (right hand) and 5.34 ± 1.52 mm (left hand), while Junior residents demonstrated baseline deviations of 5.09 ± 1.58 mm (right hand) and 5.54 ± 1.39 mm (left hand). When analyzing only the resident groups across all timepoints, right-hand accuracy improved from 5.04 ± 1.6 mm at baseline to 4.71 ± 1.1 mm at two weeks follow-up, whereas left-hand accuracy remained relatively stable (5.40 ± 1.62 mm to 5.38 ± 1.04).

### 3.3. Association Between Training and Drilling Accuracy

Among senior residents, A significant overall effect of time on right-hand accuracy was observed (F(2,14) = 5.85, *p* = 0.014), with deviation from the intended target decreasing from baseline to post-training (4.80 ± 0.78 mm) and further at the 2-week follow-up (4.71 ± 1.10 mm). Post hoc pairwise comparisons with Bonferroni correction demonstrated a trend toward improvement from baseline to post-training (*p* = 0.06) and from post-training to the 2-week follow-up (*p* = 0.105). For left-hand accuracy among senior residents, no significant overall effect of time was observed (F(2,14) = 0.46, *p* = 0.638), although a small numerical improvement was noted from baseline (5.34 ± 1.52 mm) to post-training (5.25 ± 0.58 mm) and to the 2-week follow-up (5.21 ± 1.10 mm).

In contrast, among junior residents, no significant effect of time was found for right-hand accuracy (F(2,16) = 0.65, *p* = 0.537), despite a numerical decrease from baseline to post-training (4.98 ± 0.54 mm) and to the 2-week follow-up (4.71 ± 1.50 mm). Similarly, left-hand accuracy showed no significant effect of time (F(2,16) = 0.19, *p* = 0.828), with values remaining stable from baseline (5.54 ± 1.39 mm) to post-training (5.40 ± 1.30 mm) and at the 2-week follow-up (5.42 ± 1.50 mm) (Table 2).

### 3.4. Influence of Hand Dominance

Hand dominance demonstrated a notable association with drilling accuracy. At baseline, left-handed participants exhibited superior accuracy with their non-dominant (right) hand compared to the non-dominant (left) hand performance of right-handed participants (*p* = 0.034). When analyzing the association of handedness among only the residents who completed all three time points, the pattern remained consistent. Left-handed residents (n = 4) maintained better non-dominant hand accuracy (5.00 ± 0.67mm) compared to right-handed residents (n = 13, 5.45 ± 1.02 mm) at baseline, and this difference persisted through the post-training (4.85 mm vs. 5.32 mm) and 2-week follow-up (4.90 mm vs. 5.38 mm) assessments (Table 2).

### 3.5. Comparison Between Experience Levels

Comparative analysis between experience groups at baseline revealed that senior residents and experts had comparable right-hand accuracy (*p* = 0.59). However, senior residents performed significantly worse than experts with the left hand (*p* = 0.038), indicating that experience-based differences may be more pronounced when using the non-dominant hand. When comparing junior and senior residents across all timepoints, no significant differences were observed in right-hand accuracy at baseline (*p* = 0.78), post-training (*p* = 0.14), or 2-week follow-up (*p* = 0.63). Similarly, left-hand accuracy showed no significant differences between junior and senior residents at any timepoint (baseline: *p* = 0.61; post-training: *p* = 0.36; 2-week follow-up: *p* = 0.42). This suggests that years of residency training alone may not significantly differentiate drilling performance without specialized training interventions.

### 3.6. Point-Specific Accuracy

Analysis of specific drilling points at baseline (n = 25) revealed that accuracy for point 4 (9 o’clock) and point 5 (3 o’clock) did not differ significantly between hands. (Point 4 (R4 vs. L4): *p* = 0.245, Point 5 (R5 vs. L5): *p* = 0.483). When analysing only the resident groups (n = 17) who completed all time points, point-specific accuracy showed that for junior residents, Point 4 accuracies improved significantly with the right hand from baseline to 2-week follow-up (*p* = 0.042), while no significant improvement was observed with the left hand (*p* = 0.374). For senior residents, neither Point 4 nor Point 5 showed significant improvements across time points with either hand, suggesting that overall improvements in mean accuracy may be driven by performance on other drilling points.

## 4. Discussion

The present study aimed to evaluate drilling accuracy among orthopedic surgeons with varying levels of experience, and to assess the association of specialized training and hand dominance. Our results demonstrated several key findings that have important implications for orthopedic surgical training and practice.

Expert surgeons consistently demonstrated superior drilling accuracy compared to both junior and senior residents at baseline, particularly with the non-dominant hand. This finding aligns with prior research suggesting that technical proficiency in surgical procedures improves with accumulated experience and deliberate practice [1,11]. Similar observations have been made in other technical domains such as arthroscopy, laparoscopy, and microsurgery, where expert performance is consistently associated with superior visuospatial integration, error recognition, and task economy. These findings support the broader theory that expertise is not merely the result of case exposure but of targeted, repetitive refinement of motor patterns in structured environments.

Interestingly, we found no significant differences in drilling accuracy between junior and senior residents at baseline, suggesting that years of residency training alone may not substantially differentiate technical drilling performance. This finding raises important questions about the development of technical skills during residency training and the potential need for more focused, skill-specific training interventions. Comparable results have been reported in simulation-based arthroscopy and fracture fixation models, where resident seniority did not reliably predict accuracy or efficiency in task completion. This suggests that traditional apprenticeship-based progression may not provide sufficient deliberate practice for skill differentiation, thereby underscoring the necessity of simulation-based curricula that directly target core psychomotor abilities.

Our results revealed a notable difference in the association of training across resident experience levels. Senior residents demonstrated significant improvement in right-hand accuracy following the training intervention, with sustained improvement at the 2-week follow-up. The persistent improvement from baseline to 2-week follow-up suggests that the training had a lasting effect on technical performance for this group. This finding suggests that surgeons at the senior resident level may be at an optimal stage of development to benefit from targeted technical training. In contrast, junior residents showed no significant improvement following training, with minimal changes in accuracy from baseline to post-training, post-training to 2-week follow-up or baseline to 2-week follow-up. The lack of measurable improvement among junior residents suggests that foundational skills may need to be developed before specialized drilling techniques can be effectively taught. This differential response to training across experience levels highlights the importance of tailoring orthopedic surgical education to the trainee’s stage of development, emphasizing basic motor and psychomotor competencies in early residency, while introducing more advanced, technique-specific instruction as residents progress. These stage-specific effects echo prior educational theory, including Ericsson’s [11] framework of deliberate practice, which emphasizes that performance gains are maximized when training intensity is matched to the learner’s current developmental capacity. Senior residents, having achieved baseline competence, appear more receptive to advanced psychomotor refinement, whereas junior residents may benefit more from foundational training modules focusing on posture, hand–eye coordination, and force modulation before advancing to drilling accuracy tasks.

Hand dominance is a relevant yet complex factor in orthopedic surgical training and performance. Although the majority of orthopedic surgeons are right-handed, left-handedness appears to be more prevalent in this field than in the general population, and left-handed surgeons often develop enhanced ambidexterity, likely as a functional adaptation to predominantly right-handed tools and environments [8,12]. In our study, this adaptability was reflected in superior non-dominant hand performance among left-handed participants compared to their right-handed peers, suggesting that left-handed individuals may cultivate more balanced bimanual control over time. This advantage persisted among residents who completed all assessment phases, with left-handed residents consistently maintaining better non-dominant hand performance throughout the training period, suggesting that this difference endures even after standard training interventions. The significant difference in left-hand performance between senior residents and experts, underscores the importance of experience in developing ambidextrous technical skills, which are particularly valuable during complex orthopedic procedures where the surgical approach may necessitate the use of the non-dominant hand. These findings mirror reports from laparoscopic and arthroscopic simulation studies, where hand dominance influenced task efficiency in complex procedures but had limited effect in basic skills. Importantly, ambidexterity has been shown to mitigate ergonomic challenges and improve adaptability when accessing angles or visualization constraints necessitate unconventional instrument use. This may have implications in orthopedic trauma and hand surgery, where drilling and fixation are frequently performed under constrained access.

The broader literature offers conflicting evidence on this topic: while Feeley et al. [13] found that hand dominance and experience improved performance in complex arthroscopic simulation tasks, Reppenhagen et al. [14] observed no significant effect of laterality in more basic tasks. These inconsistencies may reflect differences in task complexity, performance metrics, or definitions of proficiency. Collectively, these findings highlight the critical need to design surgical training models that consider hand laterality while promoting the development of bimanual skills. Given that orthopedic procedures frequently demand precise manipulation and instrument handling from multiple angles and approaches, fostering ambidexterity is particularly important. Future studies should therefore evaluate whether structured ambidextrous training, particularly modules designed for nondominant hand performance, can accelerate the acquisition of complex orthopedic skills. Such approaches may help reduce the gap between left- and right-handed surgeons, enhance adaptability in the operating room, and ultimately improve patient safety and surgical outcomes.

The analysis of specific drilling points (4 and 5) at baseline, revealed no significant differences in accuracy between hands, suggesting that the inherent difficulty of certain drilling tasks may normalize performance differences between dominant and non-dominant hands. However, when examining changes over time among residents, we found that point 4 accuracies improved significantly with the right hand for junior residents from baseline to follow-up, while no such improvement was observed with the left hand or for other drilling points. This point-specific improvement suggests that certain technical aspects of drilling may be more amenable to improvement through training than others. Focusing on these specific points provides insight into the nuanced aspects of skill acquisition that might be overlooked when only mean accuracy is considered. These results highlight the potential value of targeted training, emphasizing that certain technical challenges, such as precision at “point 4”, can be selectively addressed to maximize learning outcomes. Moreover, the findings inform the design of surgical training programs by suggesting that interventions tailored to the most critical points of performance may be more effective than generalized practice. Even with a relatively small sample in this preliminary study, the point-specific data offers practical guidance for optimizing hands-on training and developing ambidextrous proficiency.

Recent technological advances have explored the use of augmented reality (AR) to improve technical precision in orthopedic procedures. Van Gestel et al. [15], demonstrated that AR guidance significantly improved drilling accuracy across all experience levels, particularly in complex oblique trajectories, compared to freehand and proprioception-guided techniques. Interestingly, surgeon experience did not significantly influence performance, underscoring the tool’s broad utility. While such high-tech solutions show promise, they often require costly equipment, infrastructure, and validation in clinical settings. In contrast, our findings support the value of low-cost, simulation-based training models. These models remain accessible and scalable across institutions and may serve as a practical complement or precursor to more advanced technologies. This study offers initial insights into factors influencing drilling accuracy, while highlighting the importance of extending future research to evaluate long-term skill retention and real-world surgical outcomes.

### Strengths and Limitations

The strengths of this study include its prospective design, standardized assessment protocol, and inclusion of surgeons with varying levels of experience. The use of synthetic bone models provided a consistent medium for evaluation, minimizing variability in bone density or quality that might affect drilling accuracy.

Several limitations should be acknowledged. The sample size was relatively small, which limits the generalizability of findings. The current study was conducted with a limited number of participants, reflecting the total pool of orthopedic surgeons available at our center. Consequently, the sample size may not provide sufficient power to detect small differences between groups. We acknowledge that larger multicenter studies are necessary to validate and generalize these findings.

While the Shapiro–Wilk test was used to assess data normality, the small sample size limits the statistical power and the ability to formally confirm normality for each individual measure. Future studies with larger cohorts should perform full normality testing for each outcome measure and consider non-parametric analyses (e.g., Mann–Whitney or Wilcoxon tests) to confirm these findings. Despite this limitation, the study provides preliminary evidence of the concept using a simple and feasible training model. *p*-values near the significance threshold should be interpreted cautiously, as results are exploratory and require confirmation in larger studies. Although the study did not include a formal test–retest reliability assessment, the measurement protocol was carefully standardized. Future research should incorporate reliability testing to further validate the method and ensure reproducibility of the results. Although left-handed participants showed relatively better non-dominant hand performance, the small size of this subgroup (n = 6 overall; n = 4 among residents) limits the strength of conclusions regarding handedness. The study was conducted at a single institution, which may not represent the broader population of orthopedic surgeons. While synthetic bone models provide standardization, they may not fully replicate the complexity and variability of human bone encountered in clinical practice. Expert surgeons participated only in the baseline assessment, limiting our ability to evaluate the association with training, across all experience levels. All measurements were performed by a single, experienced investigator who reviewed all data across participants, providing high internal consistency. However, formal inter-rater reliability was not assessed, and future studies could include multiple raters to further confirm measurement precision and reproducibility. Finally, the 2-week follow-up period may not be sufficient to assess long-term skill retention.

## 5. Conclusions

This study suggests that drilling accuracy in orthopedic surgery is influenced by experience level, targeted training, and hand dominance. Expert surgeons exhibit superior precision, especially with their non-dominant hand, setting a benchmark for technical skill. Improvements seen in senior residents after focused training suggest that this group benefits most from specialized instruction, whereas junior residents may require training approaches tailored to their developmental stage. Future research should focus on developing and validating targeted interventions to enhance drilling accuracy and ambidexterity across all levels of surgical experience.

## Figures and Tables

**Table 1:** Participant demographics.

	Junior Residents (n = 9)	Senior Residents (n = 8)	Experts (n = 8)	Total (n = 25)
Dominant Hand, n (%)				
Right-handed	7 (78)	6 (75)	6 (75)	19 (76)
Left-handed	2 (22)	2 (25)	2 (25)	6 (24)
Session Completion, n (%)				
Baseline	9 (100)	8 (100)	8 (100)	25 (100)
Post-training	9 (100)	8 (100)	0 (0)	17 (68)
2-Week assessment	9 (100)	8 (100)	0 (0)	17 (68)

**Table 2:** Summary of key statistical comparisons.

Comparison	Right Hand (Mean ± SD)	Left Hand (Mean ± SD)	*p*-Value
Between Groups at Baseline (n = 25)			
Junior vs. Expert	5.09 ± 1.58 vs. 4.75 ± 0.91	5.54 ± 1.39 vs. 4.55 ± 0.79	0.132 (R), 0.087 (L)
Senior vs. Expert	4.99 ± 1.41 vs. 4.75 ± 0.91	5.34 ± 1.52 vs. 4.55 ± 0.79	0.59 (R), 0.038 (L)
Junior vs. Senior	5.09 ± 1.58 vs. 4.99 ± 1.41	5.54 ± 1.39 vs. 5.34 ± 1.52	0.78 (R), 0.61 (L)
Hand Comparison (All Participants, n = 25)			
Point 4 (R4 vs. L4)	4.87 ± 1.21 vs. 4.93 ± 1.18	–	0.245
Point 5 (R5 vs. L5)	4.78 ± 1.25 vs. 4.82 ± 1.19	–	0.483
Hand Dominance (All Participants, n = 25)			
Left-handed vs. Right-handed (non-dominant hand)	5.00 ± 0.67 vs. 5.45 ± 1.02	–	0.034

SD; standard deviation, R; right hand, L; left hand.

## Data Availability

The data presented in this study are available on request from the corresponding author.
